# Virtual Vernier Effect-Enabled Parallel Dual-Cavity Sensor for Temperature and Humidity Synchronization

**DOI:** 10.3390/nano15181427

**Published:** 2025-09-16

**Authors:** Yuting Li, Xiaoguang Mu, Yuqiang Yang, Han Xia, Yuying Zhang, Chengyu Mo, Zhihao Huang, Yitong Li, Fujiang Li

**Affiliations:** 1 Guangdong Provincial Key Laboratory of Intelligent Equipment for South China Sea Marine Ranching, Guangdong Ocean University, Zhanjiang 524088, China; 2 Research Center of Guangdong Smart Oceans Sensor Networks and Equipment Engineering, Guangdong Ocean University, Zhanjiang 524088, China; 3 Information System Integration Center of China Mobile Group Shandong Co., Ltd., Jinan 250101, China

**Keywords:** optical fiber sensor, Vernier effect, temperature and humidity measurement, optical fiber Fabry–Perot interferometer

## Abstract

This paper presents a high-sensitivity temperature and humidity synchronous measurement sensor based on virtual Vernier demodulation, designed to overcome the limitations of traditional sensors in high-sensitivity and synchronous measurements. By combining a dual-cavity parallel structure with the Virtual Vernier effect (VVE), two interferometers were designed, with one using a temperature-sensitive material (polydimethylsiloxane, PDMS) and the other using a humidity-sensitive material (polyvinyl alcohol, PVA) for temperature and humidity measurement, respectively. Based on actual interference spectra, a modulation function was used to generate the virtual reference interferometer spectrum, which was then superimposed with the sensing interferometer’s spectrum to form a virtual Vernier envelope. By monitoring the displacement of the envelope, precise measurements of temperature and humidity changes were achieved. Experimental results showed a temperature sensitivity of 5.61 nm/°C and 7.62 nm/°C, a humidity sensitivity of 0 nm/%RH and −3.07 nm/%RH, and average errors of 0.64% and 1.10% for temperature and humidity, respectively, demonstrating the feasibility of the method. The introduction of the virtual interferometer effectively reduces environmental interference with the measurement results and avoids the material loss and errors associated with traditional reference interferometers. More importantly, the VVE enables dynamic adjustment of the envelope magnification, thereby enhancing the sensor’s flexibility and overcoming the structural limitations of traditional interferometers. This sensor provides efficient and reliable technological support for future environmental monitoring and climate change research.

## 1. Introduction

Temperature [1] and relative humidity (RH) [2,3] are among the most critical environmental parameters, influencing various aspects of human life, industrial production, ecosystems, and environmental protection [4,5]. Traditional methods for measuring temperature and humidity, including resistive and capacitive sensors, as well as wet-bulb thermometers, provide reliable measurements. However, they are often limited by environmental factors, such as electromagnetic interference and salt mist corrosion, which shorten sensor lifespans and require frequent maintenance and calibration. These limitations increase the complexity and cost of monitoring systems. In contrast, fiber optic sensors operate stably in extreme environments and are immune to electromagnetic interference, ensuring high precision. Their ability for remote monitoring and compact design makes them suitable for space-limited environments [6,7], positioning fiber optic sensing as an emerging and widely adopted technology in temperature and humidity detection. Beyond fiber-optic sensors, integrated photonic microresonators have also been extensively exploited for temperature and humidity sensing applications [8]. These on-chip photonic devices, encompassing polymer- or semiconductor-based microdisks, microrings, and photonic crystal cavities, exhibit high-quality (high-Q) resonant modes, whose resonant wavelengths undergo a measurable shift in response to perturbations in ambient environmental conditions. Integrated photonic microresonators exhibit sensitivity in the order of picometers per unit change, highlighting their precision in environmental sensing [9,10]. However, their manufacturing process is inherently complex, and they are susceptible to material degradation, which poses significant challenges. In contrast, fiber optic sensors offer inherent robustness and enable effective remote monitoring, making them more suitable for demanding applications.

Commonly used temperature and humidity fiber optic sensors often utilize techniques such as transverse offset fusion splicing or etching to form specific shapes [11], enabling the transmitted light to leak into the fiber cladding and interact thoroughly with the external environment. This method sacrifices structural integrity to enhance the sensor’s sensitivity. Moreover, the performance of these sensors is often improved by coating or filling the sensing units with functional materials [12,13]. In 2016, Wu’s group fused a corrosion-treated side-hole fiber with a single-mode fiber. It coated the fiber surface with a PVA film, thereby creating a fiber optic sensor capable of simultaneously measuring both temperature and humidity. The study demonstrated that, within the humidity range of 30% RH to 90% RH, the temperature sensitivity was −6.14 pm/°C, and the relative humidity sensitivity was −23.1 pm/% RH [14]. In contrast, fiber-optic sensors based on interferometric principles exhibit significant advantages in structural design, fabrication process, and multi-parameter sensing performance. In 2019, He et al. developed a fiber Bragg grating interferometer (FPI) using single-mode fiber, hollow-core fiber, and photonic crystal fiber for temperature and humidity measurements. The humidity sensitivity was 0.06273 dB/% RH, and the temperature sensitivity was 0.01064 nm/°C [15]. The same year, Li et al. combined FPI with a modal interferometer and employed Fourier transformation to achieve simultaneous measurements. The humidity sensitivity ranged from 0.52 nm/% RH to −0.20 nm/% RH, and the temperature sensitivity from 0.38 nm/°C to −0.08 nm/°C, demonstrating high integration and flexibility [16].

Building on these advances, researchers have increasingly turned to the optical Vernier effect as a promising strategy for further enhancing sensor sensitivity [17,18,19,20]. By slightly detuning and superimposing two interferometric spectra, a minute optical path difference is converted into a substantial spectral shift, resulting in sensitivity levels far exceeding those of single interferometric sensors [21,22,23,24]. For instance, our research group optimized parallel dual-cavity FPI and dual-SI structures, generating a Vernier effect between the two interferometers. This led to a 37-fold and 10-fold increase in temperature sensitivity compared to single interferometers [25,26]. Furthermore, in 2024, Nie et al. demonstrated a dual-HCC FPI in a parallel configuration that achieved a Vernier-enhanced temperature sensitivity of 628 pm/°C—approximately 41 times higher than that without Vernier tuning [27]. In the same year, Yang et al. developed a hybrid sensor combining HCF-based FPI and FMF-based MI, incorporating a reference arm to boost sensitivity. The strain and temperature sensitivities reached −13.7 pm/με and −97.7 pm/°C, respectively [28]. Chen’s group integrated an FPI with a slightly detuned Sagnac interferometer, achieving an ultra-high temperature sensitivity of 61.11 nm/°C between 30.4 °C and 34 °C, without relying on complex fabrication or insensitive reference arms [29]. Precisely matching the amplification factors of two interferometers is both essential and challenging. Achieving an effective Vernier effect requires consistent optical path differences, high spectral contrast, and stringent control over deviations in the free spectral range (FSR). Conventional Vernier configurations typically employ parallel or cascaded interferometers, where one serves as the sensing arm and the other as a stable reference. However, effectively isolating the reference interferometer from environmental disturbances is difficult, often resulting in measurement errors and system instability. Furthermore, the inclusion of a physical reference interferometer increases system complexity [30], introduces material losses, and poses additional fabrication challenges [31].

To address these issues, we propose a high-sensitivity temperature and humidity synchronous measurement sensor based on virtual Vernier demodulation. The sensor integrates PDMS and PVA into a parallel dual-cavity FPI structure. Using techniques such as modulation functions and spatial frequency transformation, a virtual reference interferometer spectrum is generated. The two interferometers are then superimposed onto the interference spectrum of the reconstructed virtual interferometer to achieve the Vernier effect. Experimental results show that within the temperature range of 35–45 °C, the temperature sensitivity is 5.61 nm/°C and 7.62 nm/°C. In the humidity range of 40% RH to 85% RH, the humidity sensitivity is 0 nm/% RH and −3.07 nm/% RH. The average measurement errors for temperature and humidity are 0.64% and 1.10%, respectively. The proposed sensor effectively suppresses environmental interference without relying on a physical reference interferometer, thereby eliminating material loss, optical path drift, and alignment errors associated with traditional configurations. Unlike cascaded or harmonic Vernier approaches that require multiple physical interferometers, the virtual Vernier effect digitally constructs the reference spectrum. By introducing modulation functions, the amplification factor of the Vernier envelope can be dynamically tuned, offering higher flexibility and adaptability beyond the structural constraints of conventional interferometers. With its low cost, simple fabrication, and enhanced robustness, this approach demonstrates strong potential for practical engineering applications.

## 2. Sensor Principle and Simulation Analysis

### 2.1. Sensor Principle

**Figure 1:**
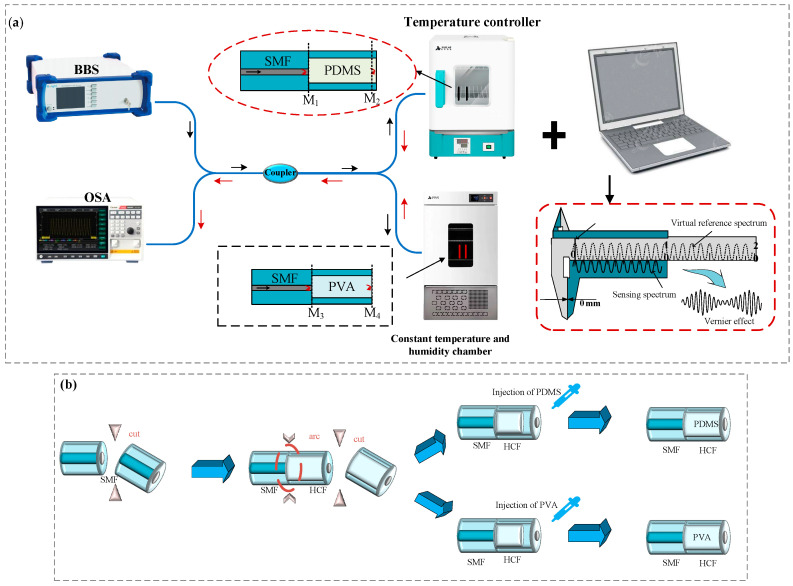
Schematic of the experimental setup and fabrication of the interferometric sensing units. (**a**) Configuration for simultaneous temperature and humidity measurements. The system consists of a broadband light source (BBS, Broadband Source), an optical spectrum analyzer (OSA, Optical Spectrum Analyzer), a constant temperature and humidity chamber (CTHC), a temperature controller furnace (TCF), and two Fabry–Pérot interferometers (FPIs). Each FPI is formed by splicing single-mode fiber (SMF) with hollow-core fiber (HCF), followed by filling with either polydimethylsiloxane (PDMS) or polyvinyl alcohol (PVA). The reflected signals are combined through an optical fiber coupler and processed by a computer to extract the sensing response; (**b**) Fabrication procedure of the sensing interferometers, showing the fusion splicing of SMF and HCF and subsequent injection of PDMS or PVA to form the sensing cavities.

We present an experimental fiber-optic sensor setup for simultaneous detection of temperature and humidity (Figure 1). This setup comprises a optical spectrum analyzer (OSA; Ceyear 6362D; Ceyear Technologies Co., Ltd., Qingdao, China; wavelength range: 600–1700 nm; maximum resolution: 0.02 nm), a broadband source (BBS; Golight Galant; Galight International, Shenzhen, China; output power: 12.80 mW; wavelength range: 1450–1650 nm; spectral power density: –17.0 dBm/nm), a Constant temperature and humidity chamber (CTHC; HWS-50B; Shanghai Yiheng Scientific Instrument Co., Ltd., Shanghai, China; humidity accuracy: 0.1% RH), and a temperature control chamber (TCF; Feisifu WGL-30B; Feisifu Instrument Co., Ltd., Wuhan, China; temperature range: RT+10–300 °C; control accuracy: 0.1 °C). A fiber coupler splits the broadband light from the source into two beams that enter FPI_1_ and FPI_2_, respectively. After reflection within each interferometer, the returning light recombines at the Optical fiber coupler and is received by the spectrometer.

FPI_1_ serves as the sensing element of the device. As shown on Figure 1b, it is formed by fusing a single-mode fiber (SMF) with a section of hollow-core fiber (HCF). At the end of the HCF, a temperature-sensitive PDMS gel is introduced via capillary action and thermally cured, creating a stable reflective surface inside the HCF. When the ambient temperature rises, the PDMS undergoes thermal expansion, increasing in volume and decreasing in refractive index. This leads to a change in the cavity length of the FPI, causing a shift in the interference spectrum. The fabrication method of FPI_2_ is identical to that of FPI_1_, except that the HCF is filled with a humidity-sensitive PVA gel. When the ambient humidity fluctuates, the hygroscopic PVA film undergoes changes in both volume and refractive index. These variations alter the effective cavity length and refractive index of the interferometer, leading to a shift in the interference spectrum and a corresponding change in the optical response of the FPI.

In FPI_1_ and FPI_2_, the reflectivity of the end faces corresponding to the PDMS (M_1_, M_2_) and PVA (M_3_, M_4_) cavities, respectively, is relatively low, allowing both to be approximated as two-beam interferometers. The interference spectral intensity can be expressed as:

(1) $$\left\{\begin{array}{l} I_{s1}\left(\lambda \right)=I_{1}+I_{2}+2\sqrt{I_{1}I_{2}}\cos\left(\frac{4\pi n_{1}l_{1}}{\lambda }\right)\\ I_{s2}\left(\lambda \right)=I_{3}+I_{4}+2\sqrt{I_{3}I_{4}}\cos\left(\frac{4\pi n_{2}l_{2}}{\lambda }\right) \end{array}\right.,$$

where *I*_1_ and *I*_2_ denote the reflected intensities from the two end faces of the PDMS cavity in FPI_1_, while *I*_3_ and *I*_4_ represent the reflected intensities from the two end faces of the PVA cavity in FPI_2_, here, *n*_1_ and *n*_2_ are the refractive indices of PDMS and PVA, respectively; *l*_1_ and *l*_2_ represent the cavity lengths of Interferometers FPI_1_ and FPI_2_, and *λ* is the wavelength of the signal light. According to Equation (1), FSR and the temperature sensitivity of the two interferometers can be derived as:

(2) $$\left\{\begin{array}{l} {\mathrm{FSR}}_{1}=\frac{\lambda^{2}}{2n_{1}l_{1}}\\ {\mathrm{FSR}}_{2}=\frac{\lambda^{2}}{2n_{2}l_{2}} \end{array}\right.,$$

The temperature sensitivities of Interferometers FPI_1_ and FPI_2_ can be expressed as:

(3) $$\left\{\begin{array}{l} S_{T1}=\frac{d\lambda_{m}}{dT}=\lambda_{m}\left(\frac{1}{l_{1}}\frac{dl_{1}}{dT}+\frac{1}{n_{1}}\frac{dl_{1}}{dT}\right)=\lambda_{m}\left(\alpha +\frac{\beta }{n_{1}}\right)\\ S_{T2}=\frac{d\lambda_{m}}{dT}=\lambda_{m}\left(\frac{1}{l_{2}}\frac{dl_{2}}{dT}+\frac{1}{n_{2}}\frac{dn_{2}}{dT}\right) \end{array}\right.,$$

Here, *α* (~9.6 × 10^−4^/°C) and *β* (~−5 × 10^−4^/°C) represent the thermal expansion and thermo-optic coefficients of PDMS, respectively. *λ_m_* denotes the peak wavelength of the interference spectrum, and *dλ_m_*/*dT* describes the wavelength shift induced by temperature changes. PDMS’s hydrophobic, non-polar molecular structure prevents hydrogen bonding or water adsorption, rendering FPI1 insensitive to humidity. In contrast, FPI_2_’s PVA gel is hygroscopic, causing its cavity length and refractive index to vary with relative humidity, altering the optical path length and shifting the resonant wavelength. This shift can be described as:

(4) $$\Delta \lambda =-\lambda_{n}\left(\frac{dl_{2}}{l_{2}}+\frac{dn_{2}}{n_{2}}\right),$$

Accordingly, the humidity sensitivity of FPI_2_ can be written as:

(5) $$S_{RH}=-\lambda_{n}\left(\frac{dl_{2}}{l_{2}dRH}+\frac{dn_{2}}{n_{2}dRH}\right),$$

In this work, we introduce a Virtual Vernier Effect (VVE) to enhance measurement sensitivity and resolution without increasing hardware complexity. A virtual reference interferometer spectrum is synthesized through signal processing techniques, leveraging modulation functions and spatial frequency transformation to reconstruct a virtual spectrum. The modulation function is:

(6) $$I_{X}=\cos\left(\frac{4\pi L_{X}}{\lambda }\right),$$

Here, *L*_X_ represents the modulation length. By setting *L*_X_ to any required value, the FSR of the virtual reference spectra can be made close to but not equal to that of the sensor interferometric spectrum. The modulation intensity obtained by multiplying the interference spectrum of the sensor interferometer:

(7) $$\begin{array}{l} I_{X}\times I_{s}=\left(I_{1}+I_{2}\right)\cos\left(\frac{4\pi l_{X}}{\lambda }\right)+\sqrt{I_{1}I_{2}}\cos\left[\frac{4\pi }{\lambda }\left(n_{1}l_{1}-L_{X}\right)\right]\\ +\sqrt{I_{1}I_{2}}\cos\left[\frac{4\pi }{\lambda }\left(n_{1}l_{1}+L_{X}\right)\right] \end{array},$$

The interference spectrum of the Virtual Reference Interferometer (VRI) is obtained:

(8) $$I_{\mathrm{VR}}=\cos\left[\frac{4\pi }{\lambda }\left(n_{1}l_{1}-L_{X}\right)\right],$$

From Equation (8), it can be known that the FSR of VRI is:

(9) $${\mathrm{FSR}}_{\mathrm{VR}}=\frac{\lambda^{2}}{2n_{1}l_{1}-L_{X}},$$

By multiplying the actual sensor interferometric spectrum *I*_s_ with the constructed virtual reference interferometric spectrum *I*_VR_, we can obtain the vernier effect envelope spectrum:

(10) $$\begin{array}{cc} I_{s}\times I_{\mathrm{VR}} & =\left(I_{1}+I_{2}\right)\cos\left(\frac{4\pi }{\lambda }\left(n_{1}l_{1}-L_{X}\right)\right)\\ & +\sqrt{I_{1}I_{2}}\cos\left[\frac{4\pi }{\lambda }\left(n_{s}L-n_{1}l_{1}+L_{X}\right)\right]\\ & +\sqrt{I_{1}I_{2}}\cos\left[\frac{4\pi }{\lambda }\left(n_{s}L+n_{1}l_{1}-L_{X}\right)\right] \end{array},$$

By superimposing the virtual reference interferometric spectrum with the interferometric spectrum of FPI_1_ and FPI_2_, respectively, two distinct VVE interference spectra can be obtained. From the second term of Equation (10), the envelope line of the vernier interference spectrum can be obtained, and the formula for their amplified FSR can be written as:

(11) $$\left\{\begin{array}{l} {\mathrm{FSR}}_{\mathrm{Envelope}1}=\frac{{\mathrm{FSR}}_{1}\cdot {\mathrm{FSR}}_{\mathrm{VR}}}{\left|{\mathrm{FSR}}_{\mathrm{VR}}-{\mathrm{FSR}}_{1}\right|}\\ {\mathrm{FSR}}_{\mathrm{Envelope}2}=\frac{{\mathrm{FSR}}_{2}\cdot {\mathrm{FSR}}_{\mathrm{VR}}}{\left|{\mathrm{FSR}}_{\mathrm{VR}}-{\mathrm{FSR}}_{2}\right|} \end{array}\right.,$$

As indicated by Equations (2) and (9), the sensitivity amplification factors of the two envelopes are:

(12) $$\left\{\begin{array}{l} M_{1}=\frac{{\mathrm{FSR}}_{\mathrm{VR}}}{\left|{\mathrm{FSR}}_{\mathrm{VR}}-{\mathrm{FSR}}_{1}\right|}\\ M_{2}=\frac{{\mathrm{FSR}}_{VR}}{\left|{\mathrm{FSR}}_{\mathrm{VR}}-{\mathrm{FSR}}_{2}\right|} \end{array}\right.,$$

The temperature sensitivity of the modulated and superimposed Vernier envelope can be expressed as:

(13) $$\left\{\begin{array}{l} S_{1}=M_{1}\cdot S_{T1}\\ S_{2}=M_{2}\cdot S_{T2} \end{array}\right.,$$

Therefore, the humidity sensitivities are given by:

(14) $$\left\{\begin{array}{l} S_{3}=M_{1}\cdot S_{H1}\\ S_{4}=M_{2}\cdot S_{H2} \end{array}\right.,$$

Here, *M*_1_ and *M*_2_ represent the sensitivity amplification factors of VVE_1_ and VVE_2_. When temperature and humidity change, the wavelength shifts in the two spectral envelopes are described by:

(15) $$\left\{\begin{array}{c} \Delta \lambda_{1}=S_{1}\Delta T+S_{3}\Delta H\\ \Delta \lambda_{2}=S_{2}\Delta T+S_{4}\Delta H \end{array}\right.,$$

where Δ*T* and Δ*H* denote variations in temperature and humidity, and Δ*λ*_1_, Δ*λ*_2_ are the corresponding spectral shifts. *S*_1_, *S*_2_, *S*_3_ and *S*_4_ are the respective temperature and humidity sensitivities of the two envelopes. The temperature–humidity sensing matrix is expressed as:

(16) $$\left[\begin{array}{l} \Delta \lambda_{1}\\ \Delta \lambda_{2} \end{array}\right]=\left[\begin{array}{ll} S_{1} & S_{3}\\ S_{2} & S_{4} \end{array}\right]\left[\begin{array}{l} \Delta T\\ \Delta H \end{array}\right],$$

### 2.2. Simulation Analysis

**Figure 2:**
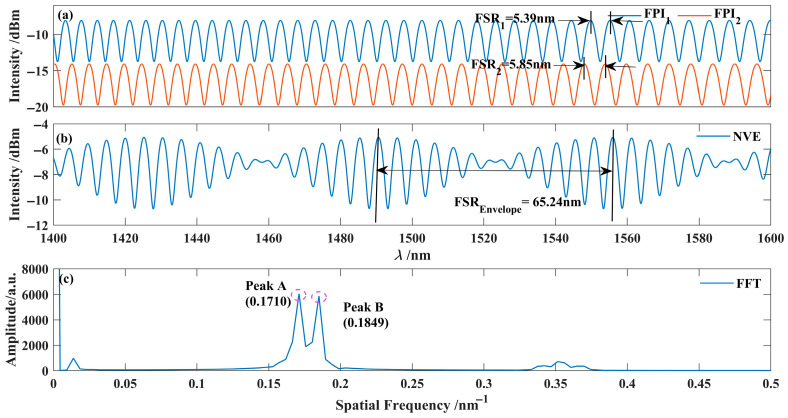
Interferometer spectrum and its Fourier transform analysis. (**a**) Interference spectra of FPI_1_ and FPI_2_ interferometers; (**b**) Parallel interference spectrum; (**c**) FFT transformation result.

We used MATLAB (R2022b; MathWorks Inc., Natick, MA, USA) to simulate the interferometric spectra of two interferometers, which were then superimposed to form the combined interference spectrum envelope. Subsequently, a Fourier transform was applied to this envelope to extract the spatial frequency information, and reconstruction simulations were performed. The simulation parameters are as follows: *I*_1_ = 0.09, *I*_2_ = 0.009, *I*_3_ = 0.09, *I*_4_ = 0.009, *L*_1_ = 158 μm, *L*_2_ = 137 μm, *n*_1_ = 1.41, *n*_2_ = 1.5, λ = 1550 nm. The interference spectra of the two interferometers were calculated according to Equation (1), and the corresponding interference spectra were obtained via simulation, as shown in Figure 2. As shown in Figure 2a, the FSR_1_ of FPI_1_ was 5.39 nm, and that of FPI_2_ was 5.85 nm around 1550 nm. After combining the interference spectra of both interferometers in parallel, the resulting spectrum envelope, shown in Figure 2b, has an FSR of 65.24 nm. Figure 2c presents the Fourier transform results of the envelope. During the spectral extraction process, two prominent frequency peaks were observed, located at 0.1710 nm^−1^ (Peak A) and 0.1849 nm^−1^ (Peak B), respectively. These peaks correspond to the interference spectra of FPI_2_ and FPI_1_, indicating that these frequency components represent the fundamental interference modes of FPI_1_ and FPI_2_.

**Figure 3:**
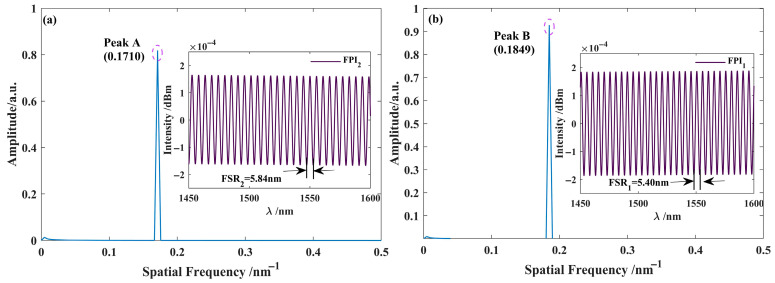
Extracted interferometric spectrum of the Interferometer. (**a**) Extracted Interferometric spectrum and frequency domain plot of FPI_2_; (**b**) Extracted Interferometric spectrum and frequency domain plot of FPI_1_.

By reconstructing the frequency domain information of the parallel interferometric spectrum envelope shown in Figure 2c, the interferometric spectra of FPI_1_ and FPI_2_ were obtained, as presented in Figure 3. The results showed that the FSR_1_ of FPI_1_ was 5.40 nm, and that of FPI_2_ was 5.84 nm, in high agreement with the simulation results (5.39 nm and 5.85 nm, respectively). This reconstruction process demonstrates high consistency with the simulated data.

**Figure 4:**
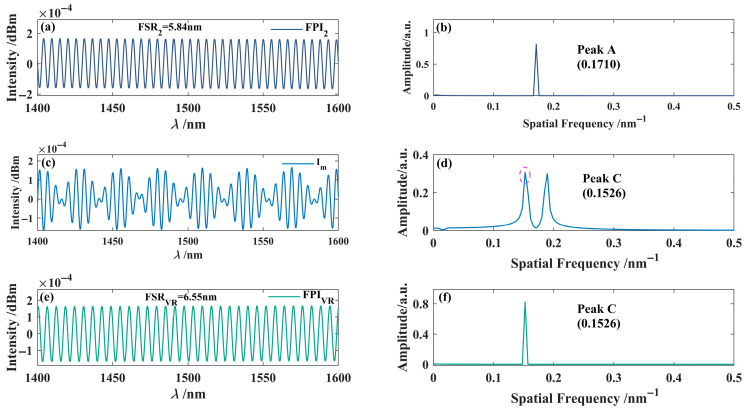
Simulated reconstruction of the interference spectrum for the virtual interferometer. (**a**) Interference spectrum of FPI_2_; (**b**) Frequency domain diagram of FPI_2_; (**c**) Interference spectrum with modulation function *I*m; (**d**) Frequency domain diagram; (**e**) The virtual reference interference spectrum generated by the first dominant peak in (**d**); (**f**) Frequency domain diagram.

In the simulation process, the cavity length *L*_X_ of the modulation function is initially set to 20 μm, and the modulation function is applied to the interference spectrum of sensing FPI_2_. The interference spectrum of the sensing interferometer is shown in Figure 4a. By introducing the modulation function, the spectrum shown in Figure 4b is obtained. After applying the Fourier transform to this spectrum, the primary frequency peaks were extracted, with the frequency-domain information of the extracted peak C (0.1526 nm^−1^) shown in Figure 4c. Furthermore, by utilizing Fourier inversion techniques, the interference spectrum of the VRI is obtained, as shown in Figure 4c. The results show that the FSR of the virtual interference spectrum is 6.55 nm, which is close to the FSRs of the two sensing interferometers (5.40 nm and 5.84 nm, respectively).

**Figure 5:**
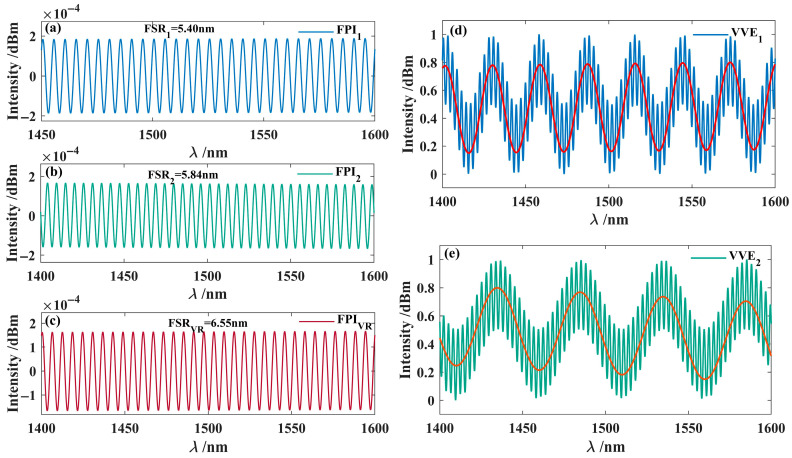
Superimposed interferometric Spectrum. (**a**) FPI_1_; (**b**) FPI_2_; (**c**) FPI_VR_; (**d**) VVE_1_, with red lines indicating the envelope of the VVE_1_; (**e**) VVE_2_, with red lines indicating the envelope of VVE_2_.

Subsequently, the interference spectra of the FPI_1_ and FPI_2_ were superimposed with that of the VRI to generate the Vernier effect, producing two interference spectrum envelopes, as shown in Figure 5. According to Equation (12), the magnifications *M*_1_ and *M*_2_ are 5.69 and 9.23, respectively. Through modulation and frequency-domain reconstruction, this method effectively extracts and superimposes interference spectra to produce a pronounced Vernier effect without requiring a physical reference interferometer. This approach demonstrates great potential for enhancing measurement sensitivity, reducing external interference, and overcoming the structural limitations of traditional interferometers.

## 3. Sensor Experimental Results and Discussion

### 3.1. Temperature and Humidity Detection Results

**Figure 6:**
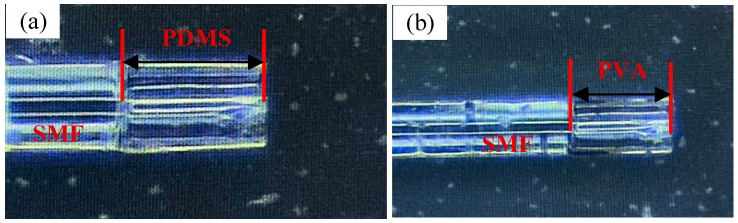
The micrographs of two interferometers. (**a**) FPI_1_; (**b**) FPI_2_.

To investigate the impact of temperature and humidity on interferometer performance, a dual-cavity parallel structure was employed, with FPI_1_ and FPI_2_ tested for their sensitivity to temperature and humidity, respectively. Figure 6 shows micrographs of the two interferometers. In FPI_1_, PDMS, a temperature-sensitive material, responds to temperature changes. Its hydrophobic properties prevent water molecules from contacting the optical fiber interior, resulting in high temperature sensitivity and insensitivity to humidity. In FPI_2_, PVA gel fills the hollow-core fiber, forming a transparent gel-like film after drying, which is sensitive to humidity due to its hygroscopic nature.

**Figure 7:**
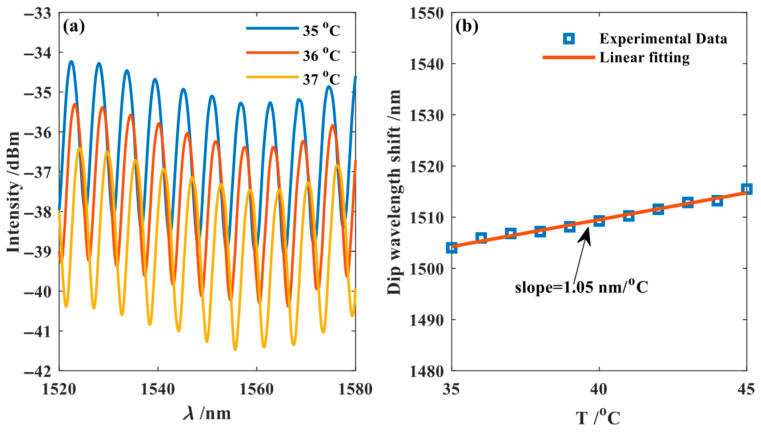
The temperature measurement result of FPI_1_. (**a**) Temperature measurement experiment results of FPI_1_; (**b**) Experimental data fitting FPI_1_.

In the temperature experiments, FPI_1_ and FPI_2_ were placed in the temperature control chamber, with the temperature range set from 35 °C to 45 °C. Interference spectra were recorded at 1 °C intervals. The temperature measurement results for FPI_1_, as shown in Figure 7, revealed a significant wavelength redshift with increasing temperature. The peak wavelength at 1550 nm was tracked, and the data were fitted. The experimental results show that the temperature sensitivity of FPI_1_ is 1.05 nm/°C.

**Figure 8:**
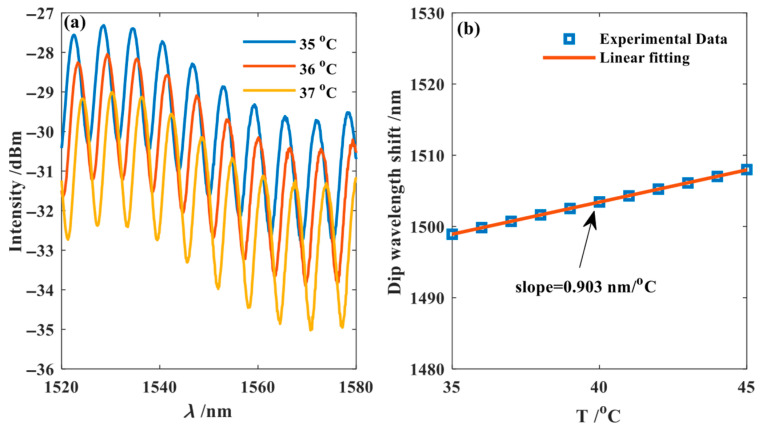
The temperature measurement result of FPI_2_. (**a**) Temperature measurement experiment results of FPI_2_; (**b**) Experimental data fitting FPI_2_.

For the temperature measurement of FPI_2_ (shown in Figure 8), the interference spectrum also shifted towards longer wavelengths. A similar fitting analysis of the peak wavelength data at 1550 nm revealed that the temperature sensitivity of FPI_2_ is 0.903 nm/°C.

**Figure 9:**
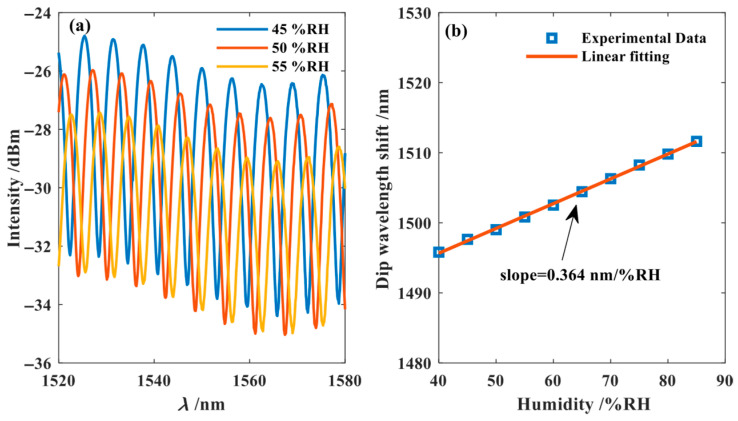
The humidity measurement result of FPI_2_. (**a**) Humidity measurement experiment results of FPI_2_; (**b**) Experimental data fitting FPI_2_.

To investigate the humidity response of FPI_1_ and FPI_2_, both were placed in a temperature–humidity chamber maintained at 25 °C. The humidity range of the chamber was set from 40% RH to 85% RH, with a step size of 5% RH. To ensure experimental stability, the interference spectrum data under different humidity conditions were recorded using the optical spectrum analyzer after allowing the humidity to stabilize with each change. The humidity sensitivity of FPI_1_ is approximately 0 nm/% RH. As shown in Figure 9, the interference spectrum of FPI_2_ exhibited a blue-shift phenomenon with increasing humidity. By tracking the peak wavelength near 1550 nm, data fitting revealed a humidity sensitivity of −0.364 nm/% RH for the FPI_2_.

**Figure 10:**
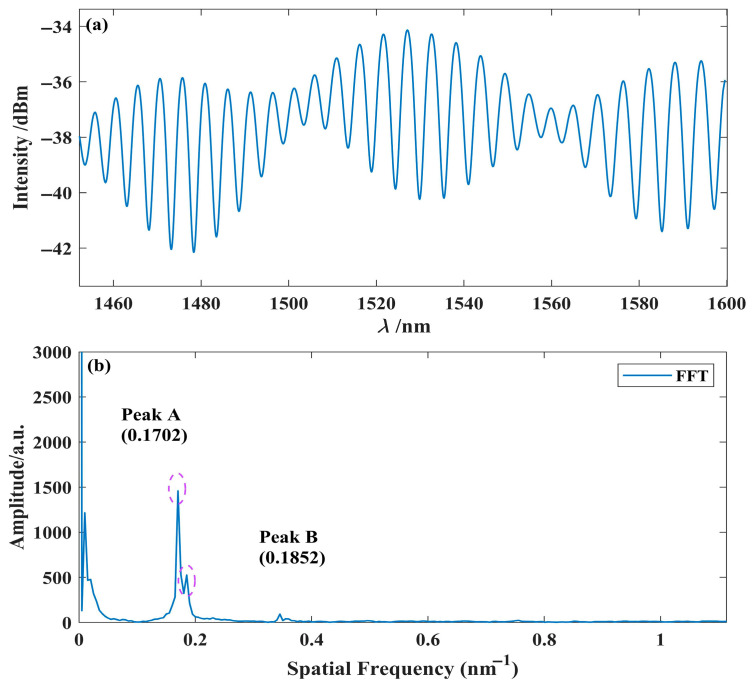
Experimental results. (**a**) Parallel interference spectrum; (**b**) the result of the fast Fourier transform.

To investigate the simultaneous temperature and humidity detection characteristics, FPI_1_ and FPI_2_ were placed in parallel in a constant temperature–humidity chamber to ensure that temperature variations did not interfere with the experimental results. The synchronous detection envelopes of FPI_1_ and FPI_2_ are shown in Figure 10a. Before introducing the VRI, Fourier transform was performed on the synchronous detection envelope to extract the independent interference spectra of both interferometers. Figure 10b shows the spatial frequency spectrum information extracted through Fourier transform, revealing two prominent peaks: the first peak occurs at 0.17025 nm^−1^ (Peak A), corresponding to the interference spectrum of FPI_2_; the second peak occurs at 0.18527 nm^−1^ (Peak B), corresponding to the interference spectrum of FPI_1_.

### 3.2. Simultaneous Temperature and Humidity Detection Results

**Figure 11:**
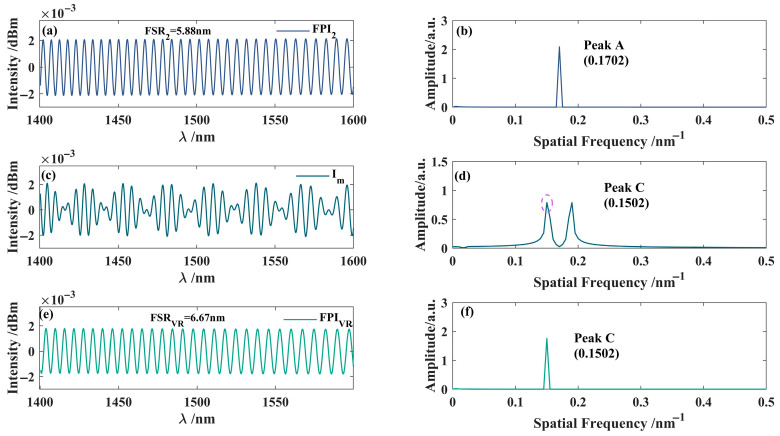
Experimental reconstruction of the interference spectrum for the virtual interferometer. (**a**) Interference spectrum of FPI_2_; (**b**) frequency domain diagram of FPI_2_; (**c**) interference spectrum with modulation function *I*m; (**d**) frequency domain diagram; (**e**) the virtual reference interference spectrum generated by the first dominant peak in (**d**); (**f**) frequency domain diagram.

To construct the interference spectrum of the virtual interferometer, it is only necessary to perform a Fourier transform on the spectrum of either sensing interferometer and apply a modulation function to shift its frequency, thereby extracting the VRI spectrum. This study generates the VRI spectrum based on the interference spectrum of FPI_2_. As shown in Figure 11, the interference spectrum of FPI_2_ in Figure 11a is first subjected to a Fourier transform to obtain Figure 11b. Subsequently, the frequency spectrum is adjusted using a modulation function to generate an interference spectrum with a modulation function, as shown in Figure 11c. The first main peak spectrum information from the FFT of this spectrum is extracted to generate the VRI spectrum in Figure 11e.

**Figure 12:**
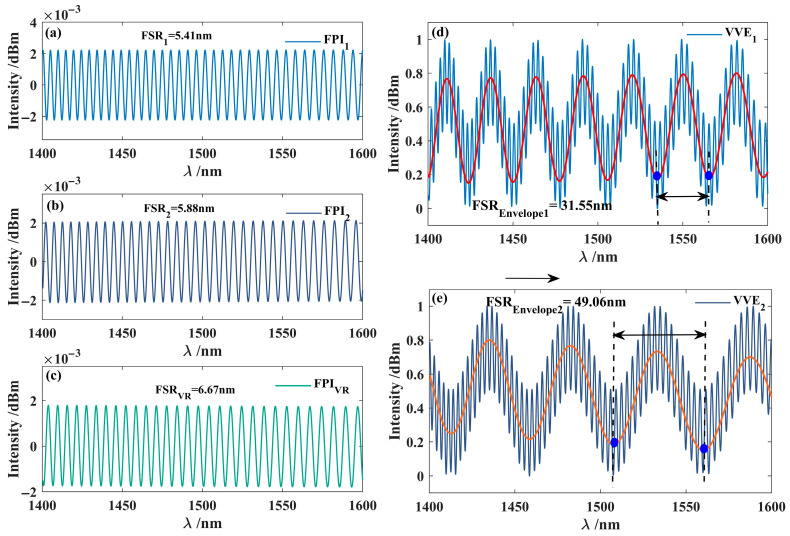
Superimposed interferometric Spectrum. (**a**) Interference spectrum of FPI_1_; (**b**) interference spectrum of FPI_2_; (**c**) virtual interference spectrum FPI_VR_; (**d**) VVE_1_, with red lines indicating the envelope of the VVE_1_(FSR_envelope1_ = 31.55 nm); (**e**) VVE_2_, with red lines indicating the envelope of the VVE_2_(FSR_envelope2_ =49.06 nm).

Figure 12 presents the extracted interference spectra of FPI_1_, FPI_2_, and the VRI. From the interference spectrum, it can be seen that the FSR values of FPI_1_, FPI_2_, and VRI are 5.41 nm, 5.88 nm, and 6.67 nm, respectively. Figure 12d shows that the reconstructed FPI_1_ is superimposed with the virtual reference spectrum, forming an interference spectrum envelope. The FSR of this envelope was measured to be 31.55 nm, with an amplification factor of 5.34. In Figure 12e, the virtual reference spectrum and FPI_2_ are superimposed to generate a second interference spectrum envelope, whose FSR is 49.06 nm and a magnification factor M_2_ of 8.44.

In the above experiment, the sensitivity of the two cavities to temperature and humidity, as well as the amplification factors of the reconstructed interference spectrum envelopes, were measured. By substituting these parameters into Equation (16), the temperature–humidity sensing matrix is expressed as:

(17) $$\left[\begin{array}{l} \Delta T\\ \Delta H \end{array}\right]={\left[\begin{array}{ll} 11.6\times 1.05 & 11.6\times 0\\ 7.2\times 0.903 & 7.2\times 0.364 \end{array}\right]}^{-1}\left[\begin{array}{l} \Delta \lambda_{1}\\ \Delta \lambda_{2} \end{array}\right]$$

When the parameters of environmental temperature and relative humidity change, the interference envelope spectrum will show a corresponding wavelength shift. By precisely tracking the peak wavelengths of the interference spectrum envelope and substituting them into the temperature and humidity sensing matrix Equation (17), the synchronous calculation of environmental temperature and humidity can be achieved. The introduction of the VVE significantly improves the measurement sensitivity of the system. Specifically, the temperature sensitivity of interference envelope 1, when humidity sensitivity is not considered, increases to 5.61 nm/°C. Meanwhile, the temperature sensitivity of interference envelope 2 is 7.62 nm/°C, with a humidity sensitivity of −3.07 nm/% RH. The sensitivities were, respectively, amplified by approximately 5.34 and 8.44 times.

Table 1 presents detailed results of the temperature–humidity matrix equation demodulation, with average measurement errors of 0.64% for temperature and 1.10% for humidity in the sensing system. The humidity error is slightly higher than the temperature error, primarily due to peak detection in the interference spectrum envelope and potential deviations in the Fourier transform process during spectrum extraction. Additionally, environmental factors such as air flow and impurities have a greater impact on humidity measurements, which may also introduce additional system errors. As detailed in Section 3.1, the hydrophobic PDMS in FPI_1_ results in negligible humidity sensitivity (0 nm/%RH), effectively isolating temperature measurements from humidity fluctuations. However, the hygroscopic PVA in FPI_2_ exhibits a temperature sensitivity of 0.903 nm/°C along with its humidity sensitivity of 0.364 nm/%RH, potentially allowing temperature changes to influence humidity readings. The temperature-humidity sensing matrix (Equation (17)) addresses this by enabling simultaneous demodulation to decouple the parameters, as demonstrated by the low average errors (0.64% for temperature, 1.10% for humidity) in Table 1, confirming reliable performance in the tested ranges.

#### Evaluation of Sensor Repeatability and Long-Term Stability

**Figure 13:**
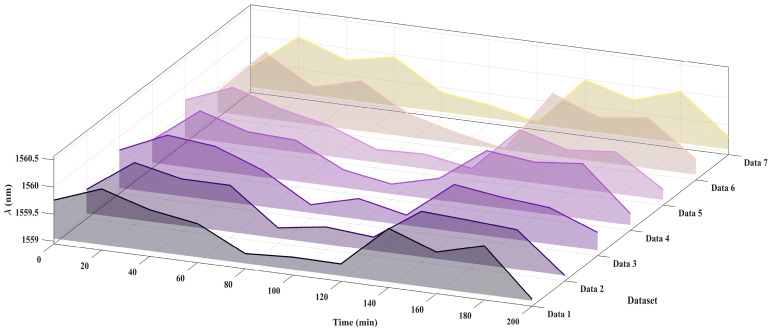
Visualization of repeatability in sensor response using a 3D waterfall plot.

To evaluate the repeatability and long-term stability of the sensor, seven consecutive measurements were performed under identical conditions. As shown in Figure 13, during a monitoring period of 200 min with data collected every 20 min, the valley position of the cascaded interference envelope near 1550 nm consistently remained within 1559.7 ± 0.5 nm. The interference spectra obtained from repeated measurements exhibited high consistency, demonstrating the excellent repeatability and stability of the sensor. These findings highlight the robustness of the proposed virtual Vernier effect-based sensing method and further confirm its reliability for practical applications.

As shown in Table 2, existing designs achieve commendable sensitivities and unique advantages, yet they often encounter practical limitations such as high insertion loss, structural complexity, fragile geometries, or costly fabrication. These drawbacks may hinder their reproducibility and large-scale deployment. By contrast, the present work introduces a dual FPI configuration employing PDMS and PVA as functional layers, in combination with a digitally constructed virtual Vernier reference. This strategy eliminates the dependence on a physical reference interferometer, thereby mitigating issues of material loss, optical path drift, and alignment errors. Furthermore, the modulation-based design allows dynamic tuning of the Vernier envelope magnification, offering flexibility beyond the structural constraints of conventional sensors. Consequently, the proposed method delivers high sensitivity, reduced cross-sensitivity, and enhanced adaptability, demonstrating clear advantages over existing state-of-the-art fiber optic temperature and humidity sensors. At the same time, this method inevitably involves trade-offs, namely higher computational complexity, potential latency from spectral reconstruction, and performance dependence on the Vernier magnification factor, requiring appropriate modulation length selection to balance sensitivity.

## 4. Conclusions

This study presents a high-sensitivity simultaneous temperature and humidity measurement sensor based on virtual Vernier demodulation. The virtual interference spectrum is generated by using the modulation function, and the two interferometers are, respectively, superimposed with it to produce the VVE. By tracking the shift in the interference spectrum envelope, a sensitivity coefficient matrix was constructed, enabling synchronous temperature and humidity detection through matrix equation demodulation. The experimental results show that the temperature sensitivity of the envelope1 is 5.61 nm/°C, while envelope2 exhibits a temperature sensitivity of 7.62 nm/°C and a humidity sensitivity of −3.07 nm/% RH, with envelope1 showing negligible humidity sensitivity (0 nm/% RH). The average measurement errors, derived from the temperature-humidity matrix equation, are 0.64% for temperature and 1.10% for humidity. This sensor generates a virtual interference spectrum using modulation functions, the sensor effectively reduces environmental interference without requiring a physical reference interferometer, thus avoiding material losses and errors. The dynamic adjustment of the Vernier envelope’s magnification factor enhances flexibility, overcoming the limitations of traditional interferometers. It shows significant potential for environmental monitoring and climate change research.

## Figures and Tables

**Table 1:** Demodulation results of temperature and humidity and measurement results.

Serial Number	Temperature Demodulation Result (°C)	Temperature Measurement Result (°C)	Error 1(%)	Humidity Demodulation Result (%RH)	Humidity Measurement Result (%RH)	Error 2 (%)
1	35.079	35	0.23	39.581	40	1.05
2	35.477	36	0.89	45.469	45	1.04
3	36.165	37	0.63	49.304	50	1.39
4	37.313	38	0.81	54.365	55	1.15
5	38.411	39	0.98	59.343	60	1.09
6	39.488	40	0.29	64.139	65	1.32
7	40.443	41	0.62	69.544	70	0.65

**Table 2:** Comparison of representative fiber optic temperature and humidity sensors.

Structure and Materials	Temperature Sensitivity	Humidity Sensitivity	Key Features	Ref.
Side-hole fiber coated with PVA	−6.14 pm/°C	−23.1 pm/%RH	High sensitivity; high loss; poor stability	[14]
FPI with PCF + hollow-core fiber	0.01064 nm/°C	0.06273 dB/%RH	Low cross-sensitivity; fast response; complex fabrication	[15]
Cascaded FPI + MI, Fourier demodulation	0.38 nm/°C and −0.08 nm/°C	0.52 nm/%RH and −0.20 nm/%RH	High integration; flexible demodulation; heavy computation	[16]
FBG + CMC/CNT film on hollow-core fiber	0.0263 nm/°C	0.1705 nm/%RH	Hybrid design; reduced crosstalk; costly coating	[32]
Dual D-shaped fibers with toluene and PVA coatings	1.02 nm/°C	0.79 nm/%RH	High sensitivity; FEM optimization; expensive fabrication	[33]
U-shaped microfiber with MoS_2_ coating	−0.013 nm/°C	0.116 nm/%RH	High sensitivity; fast response	[34]
Dual FPI (PDMS + PVA) with Virtual Vernier Effect	7.62 nm/°C	−3.07 nm/% RH	High sensitivity; Reduced cross-sensitivity; no physical reference cavity; low cost; dynamically tunable M factor	This work

## Data Availability

The raw data supporting the conclusions of this article will be made available by the authors on request.

## Abbreviations

VVE	Virtual Vernier Effect
VRI	Virtual Reference Interferometer
